# Principles of General and Comparative Physiology

**Published:** 1842-01

**Authors:** 


### Art. VIII.

*Principles of General and Comparative Physiology.* By W. B. Carpenter, m.d. Second Edition.—*London*, 1841. 8vo, pp. 577.

It affords us the utmost pleasure and satisfaction to find ourselves so early called upon to notice a second edition of this work. We principally rejoice in this because we most conscientiously believe that the extensive diffusion of the physiological views and doctrines which it contains will not only powerfully tend to the advancement of medical science, but will give a higher tone and more philosophical cast to the general doctrines of physiology. It appears that before the time of Hippocrates medicine formed a branch of philosophy, and Socrates, Plato, and their immediate disciples devoted considerable attention to the study of anatomy and physiology. The anatomico-physiological descriptions of Plato and Zenophon have been quoted by Longinus as specimens of the sublime. The work before us will, we have no doubt, by its clear, concise, yet comprehensive and logical exposition of the doctrines of physiology, largely contribute to restore that science to the place it formerly justly held in the estimation of those who devote themselves more exclusively to the moral and physical sciences. If these subjects, in the crude and imperfect condition in which they existed among the ancients, were thought worthy of the careful attention of the sages of ancient Greece, how much more deserving are they, in their present improved state, of the attention of those superior minds, whose mental energies disdain to be bounded within the narrow circle of one science, and who delight in extensive and connected views of nature's works? The general doctrines of physiology are eminently fitted to improve and discipline the mind; and there are not to be found in the whole circle of the sciences subjects more deserving of the consideration of the rational and intelligent mind of man than those embraced by the laws which regulate the organic development of animals and vegetables. We hope the time is fast approaching when lectures on comparative anatomy and physiology will form a part of every well-regulated system of general education. As some of the most important doctrines in the favorite science of geology are now founded upon the structure and physiological conditions of the remains of those animals and vegetables found imbedded in various parts of the strata forming the external crust of the earth, those who may wish to make any satisfactory advances in a knowledge of that science would find their progress much facilitated by a careful perusal of the contents of this volume. It was intended by the author that his work should be equally adapted for the general and the professional student, and in this he has admirably succeeded.

The grounds upon which we maintain the great excellency of this work, and so earnestly recommend it to our professional brethren, may be shortly stated.

1. We are convinced that philosophical physiology must be founded upon an examination of the vital actions of all organized bodies, vegetables as well as animals, and that it is equally absurd to attempt to ascertain physiological laws from a consideration of one class only of organized bodies, as it would be to lay down the characters of a particular nation or tribe of men from an examination of one only of the numerous individuals of which it is composed. This doctrine is not only inculcated, but fully followed out by our author.

2. We believe that it contains just and correct views regarding the *nature and cause of vital action.* The proper signification of the term *life* is the assemblage of the characteristic phenomena manifested by an organized structure, and by the term *vitality* is meant the quality or property of organized bodies of exhibiting certain phenomena, the sum of which constitutes *life.* The expression *vital actions* may in the same manner be used simply to express those phenomena which occur in organized living bodies, which cannot at present be referred to chemical or physical laws, and without the slightest reference to any theoretical notions as to the cause of these actions. The chief objection to the doctrine of a vital principle is not that we can prove it to be false, not that it cannot possibly be true, but that it is highly unphilosophical to assume the existence of an agent of which there is not the slightest proof, the subject being in fact of such a nature as not to admit of any satisfactory evidence. We believe as thoroughly as any vitalist can do, that many of the phenomena of living bodies cannot and never will be explained by chemical and physical laws; we strongly and earnestly disclaim the slightest participation or wish to convert the human body into a chemical or mechanical apparatus; we boldly proclaim the divine origin of all organized bodies; but we do deny, on all the principles of induction and reasoning, that any one is entitled to assert that the phenomena peculiar to living bodies are the result of any single or of any particular set of agents or entities, whether material or immaterial.

When we examine the objects of the inorganic kingdom, we find that they exhibit certain physical properties which we term hardness, extension, &c., that they manifest certain actions called attraction, chemical affinities, &c. On the other hand, when we investigate organized bodies, we perceive that besides exhibiting various actions similar to those which we witnessed in the inorganic world, they also manifest certain actions peculiar to themselves, to which we give the name of nutrition, muscular contraction, &c., so that, proceeding on philosophical principles, we divide all the objects on the earth's surface into two great groups, into those which are organized and into those which are unorganized, these being distinguished from each other by wide and important differences in their structures and qualities. It is only on account of these differences in their qualities that we are entitled to separate physiology from natural philosophy; for if the manifestations of activity in an organized body, which we are at present obliged to refer to *muscular contractility*, &c., had any bonds of affinity with those which we witness in inorganic matter, physiology would then form a part of natural philosophy. All our knowledge of the inorganic kingdom consists in an acquaintance with the qualities and actions which it embraces, and all our knowledge of organized bodies is also derived from a similar examination of their qualities and actions, and it would just be as reasonable and equally logical to refer all the actions exhibited by inorganic matter to some presiding *anima mundi*, as it is to call in the agency of a vital principle to account for the manifestation of activity in organized structures. The greatest length to which our finite faculties can carry us in the investigation of either organized or unorganized bodies, is to trace up effects, if possible, to what are called their causes, and to refer them as far as we are able to some of the great properties with which the Creator has endowed matter; for in every science we arrive at certain great facts beyond which we have but little prospect of passing, such as *attraction* in the physical sciences, and *muscular contractility* in physiology; and it is impossible to conceive how the religious faith of that man who believes that life depends upon certain properties impressed upon matter by the hands of its Creator,—properties cognizable by their effects on the senses, and whose existence is capable of demonstration,—can be weaker than his who asserts that life depends upon some unknown entity conjured up by his own imagination. Some of the vitalists (as our author has himself experienced) have not hesitated in their zeal for their favorite doctrine, to raise the cry of materialist and atheist against all who refuse to adopt their dogmas. It may appear to some that the differences between the vitalists and those who entertain opinions similar to our author, are but trifling after all, and not worth the disputing about; for while the vitalist refers the phenomena peculiar to organized bodies to an imaginary entity, their opponents refer them to certain properties of organized bodies which they designate by the term *vital properties;* but upon reflection it will be found, that the assumption of an imaginary *vital principle* is not only totally uncalled for, but that the recognition of such supposed entities has exercised a most baneful influence upon the progress of science. It has done this, not only by leading the mind into discussions upon things which can never be proved to exist, and of whose nature—even admitting that they do exist—we can know nothing, but also by perpetuating that vagueness and indistinctness which must necessarily float around our notions of the agency of an unknown immaterial principle. Accordingly it was not until natural philosophers had discarded all such entities from their discussions, and applied themselves to the observation and the classification of phenomena, that such brilliant discoveries rewarded their labours.

3. We believe that our author entertains very correct notions upon the agency of the capillary blood-vessels, in the performance of the functions of circulation, nutrition, and secretion. It must be known to all our readers that since the time of Cullen, many most important phenomena, both in the healthy and morbid conditions of the body, have been attributed to the actions of the coats of the capillary blood-vessels. The regulation of the supply of blood in particular organs and tissues, the functions of nutrition and secretion, the phenomena of inflammation, &c. have been, and by many still are, chiefly or entirely referred to the contractions and relaxations of the capillary blood-vessels. In these opinions are contained many grave and serious errors, which must be entirely discarded before any very important advances can be made, either in physiology or in pathology. Such errors could never have arisen if physiologists had not formerly restricted their investigations almost entirely to the vital actions of man and the higher organized animals. A stronger argument could not be produced in favour of the plan of studying physiology adopted in the work before us, than the important light which it throws upon these subjects. There can be no doubt that the functions of nutrition and secretion may take place without the agency of vessels, and that when present they merely answer the purpose of *carriers*, for the more abundant and ready transmission of the nutritious fluids. In the same way the regulation of the supply of blood in particular organs and tissues depends not upon the contractions of the coats of the capillaries, but upon certain actions and reactions which go on between the blood and the surrounding tissues. The phenomena of inflammation and the formation of morbid products and growths do not depend upon the capillaries, and we are fully satisfied that those who seek for their explanation in a particular arrangement of the capillary vessels are entirely in a wrong track. We do not doubt that minute injections of morbid growths may disclose some interesting facts, and may form one of the elements by which we may ultimately be enabled to arrive at a knowledge of the general laws regulating their production; but to continue to attach the importance to this mode of investigation which some pathologists still do, argues a want of accurate information on the subject.

4. We believe that our author is also perfectly correct in maintaining that nutrition, secretion, and muscular contractility, are properties inherent in organized tissues, and are not derived from the nervous system, although they may be influenced by causes acting through it. Those who examine the manifestations of activity in all organized bodies can have little difficulty in arriving at this conclusion.

In a former Number (Vol. VII. p. 168,) we gave an analysis of some of the most important chapters of the first edition of this work, and expressed our highest admiration both of the extent and accuracy of the information which it contained, and the perspicuity and conciseness of the style in which it was written. We need not again point out the general scope and character of the work, and shall therefore confine our attention to the additions and improvements which are contained in this edition. From the free and rapid communication which now exists between the different nations of Europe, and from the numerous and talented investigators everywhere at work, facts and observations are accumulating in medical science, as in every other science, with a celerity hitherto unknown in the history of the world; and our author has consequently had to *modify* certain passages of the former edition, and to incorporate some new and important matter with the old. The most valuable additions will be found in the chapters on "the primary tissues of organized bodies;" on "the special and comparative physiology of nutrition;" and on "the reproduction of organized beings." In the former edition the author, when treating of the elementary tissues of vegetables, arranged them into three groups: 1, Cellular tissue; 2, Woody or fibrous tissue; 3, Vascular tissue. In this edition to these he adds a fourth kind, the Laticiferous tissue. This "consists of a series of branched tubes, anastomosing with each other, so as to form a network, in which the latex or elaborated sap flows. It is in this branching character that it chiefly differs from other forms of tissue, which never exhibit anything approaching to it." As the author remarks, "the resemblance of this system of vegetable vessels to the capillary vessels in animals is very striking." In entering upon the examination of the primary tissues of animals, our author is enabled by late microscopic researches, for which we are chiefly indebted to the German physiologists, to show that "certain animal tissues permanently retain the cellular form, and thus constitute a close *approximation* to the parenchyma of plants. Among these we have not only fat, but also the *chorda dorsalis,* or gelatinous column which replaces the bodies of the vertebræ in some of the lowest fishes, and which occupies for a time the same position in the embryos of all higher vertebrata; the epidermic tissue and its appendages, such as the hoofs, nails, claws, scales, the medulla of the stem of feathers, &c.; and to a greater or less extent, in some kinds of cartilage." He fails not also to notice that "it is now satisfactorily ascertained that the substance of the tooth is formed, like that of bone, by the deposition of calcareous matter in cells and tubes, which originally present the ordinary characters of cellular parenchyma; but it does not, like bone, pass through the intermediate stage of cartilage."

In describing those portions of the muscular tissue in which the fibres present the transverse striæ or beaded appearance, and those in which the fibres present neither the longitudinal nor the transverse striæ, and are more flattened and smaller, Dr. Carpenter might have more clearly pointed out their functional differences. He speaks of the former structure as belonging to the voluntary, and the latter to the organic muscles. Now it could readily be shown that this structural difference does not depend upon the circumstance of the muscle being called into contraction by the influence of volition, or in some other way; but upon the mode in which the contraction manifests itself; or, to use more correct language, this structural difference in the muscular fibre causes a difference in its contractile properties, altogether unconnected with the circumstance of its forming part of an organ of voluntary or of involuntary motion. These muscles, which can contract and relax rapidly, possess the former structure; those which contract and relax slowly and in a vermicular manner, present the latter structure. As the first kind of contraction could be alone useful in an organ of voluntary motion, all the muscles placed under the influence of volition possess the former structure; but as there are certain rapid muscular movements required in the involuntary or organic functions of the body, such as the contractions of the heart, and those of the pharynx in deglutition, all the muscles of organic life do not present the latter structure. The muscular fibres of the pharynx present the striated or beaded appearance, and those of the heart also possess these striæ, though somewhat indistinctly. It is more probable that the slight structural differences between the muscular fibres of the heart and those of a voluntary muscle are connected with the differences of the mode in which their contractile properties manifest themselves, than with the circumstance that the one acts involuntarily and the other voluntarily. If we could agree in designating that kind of contractility which manifests itself by rapid contractions and rapid relaxations, *muscular contractility*, as occurring in the most characteristic parts of the muscular tissue; and that which manifests itself by slow contractions and slow relaxations, as the stomach and intestines, *simple contractility;* we could then state that the muscles which have the property of *muscular contractility* present the striated appearance, while those which have the property of *simple contractility*, show no longitudinal nor transverse striæ, and are smaller and more flattened. Whether this phraseology be adopted or not, it is obvious that the terms voluntary and organic muscles used with a reference to these structural differences, may lead to error. In speaking of the *nervous tissue*, our author adopts the opinion of Remak and others, that there are small gray filaments in the sympathetic system which have been termed *organic.* We formerly stated our belief in the existence of these organic filaments, but as the question may still be considered as sub judice, we shall not at present enter upon it. In the section on the " transformation of tissues," among other highly interesting remarks, the author directs our attention to the fact, that morbid growths not unfrequently present themselves, which may be resolved into elements resembling those of the proper structures of the body; " and that the peculiar character of *malignity* which the surgeon assigns to these growths should be founded on their rapid increase, their destruction of the tissues amongst which they are developed, and their tendency to make their appearance in other parts of the body, even after the removal of the original diseased growth, rather than on any essential peculiarity in their ultimate organization, or in their chemical composition. The presence of cells or vesicles, containing nuclei, the walls of which cells are easily separable from one another, is an almost constant characteristic of these growths, and thus we may regard them as not having advanced far in the process of organization. These cells frequently become elongated, so as to present the *spindle* form (like that of the woody tubes), and when such cells are regularly arranged in clusters the tissue will possess more or less of a fibrous character. In no instance has the complete transformation of the cell itself into a bundle of fibres been observed; so that the whole tissue may be regarded as developed upon the low and simple type of the vesicular tissue of the lower plants and animals." As this last observation is meant by the author to apply only to morbid growths, we admit that it is, as far as we are aware, perfectly correct; but there can be no doubt that some of the non-malignant growths, though cellular at first, afterwards assume a distinctly fibrous appearance, and it is in all probability on this circumstance that their non-malignancy depends.

In the chapter on the " nutrition and formation of tissues," our author has given a clear and concise account of the very interesting and important researches of Schleiden, Schwann, and their followers on these subjects, and has pointed out certain modifications which, in his opinion, require to be made in Schleiden's account of the formation of the primitive cells of vegetable tissue, and that of Schwann of the formation of the primitive cells of the animal tissue, in consequence of the results of more recent researches. Our space will not permit us to enter upon this question, and we shall content ourselves by indicating it to our readers.

The progressive development of the various tissues of all organized bodies apparently from a simple glutinous fluid in which cells present themselves, capable of generating new cells, and in other cases also of undergoing various transformations by which they assume the fibrous, tubular, and lamellar structures, characteristic of certain tissues when fully formed, is a subject of extreme interest, and when fully cleared up may lead to most important consequences. Until we have ascertained the laws which regulate those molecular movements by which nutrition and secretion are effected, we can never hope to ascertain the nature and origin of those numerous structural changes in the textures of the body which pass under the various names of tubercles, tumours, &c., and which evidently depend upon some alteration of these natural molecular movements by which the materials are deposited in the different tissues of the body for their nutrition, or eliminated from the common mass of the blood by the various secretory organs. If once these laws could be ascertained,—and if this is ever to be done, it is not by making minute vascular injections alone, but by following in the track which the researches of Schleiden and Schwann have opened up to us,—then we might hope to be able to devise some more effectual method of controlling those diseased actions, by which morbid growths and depositions might be arrested; and many of those mutilations of the body by the knife of the surgeon—those opprobria of surgery, as John Hunter used to call them—which at present are absolutely necessary, may be prevented. When facts and observations shall have accumulated on these subjects, we indulge in the fond hope that in the process of time some future Harvey will arise who, by his successful generalization, will give another mighty impulse to the healing art, such as was given to it by the discovery of the circulation of the blood.

We are glad to find that our author has much extended his chapter on the "Reproduction of organized beings," and made it proportionate to the rest of the work. In the execution of this change he has amply succeeded in showing that "the philosophical pursuit of such an enquiry may be perfectly consistent with the purest delicacy of feeling, so that the general as well as the professional reader may enter upon it without reserve." No department of physiology has received so many additions within the last few years as that connected with the reproduction and development of organized bodies. It has now assumed a more definite and philosophical character, forming a marked contrast to the vague and hypothetical assumptions of which it was until lately composed. Our author has as usual collected from all quarters the numerous and scattered facts bearing upon this subject, and presented them in a faithful and lucid manner to his reader. From the extended comparisons into which he has been led between the processes of reproduction as performed by different classes of organized beings, he has arrived at a very interesting and original series of analogies, which will greatly simplify our knowledge on these questions, and much facilitate its acquisition. In detailing his views he first takes as his basis two facts lately ascertained. 1. "All organized tissues take their origin in cells, and the ordinary mode of increase of cells (whether animal or vegetable) is by the development of new cells within them, from the granular germs which they contain." 2. It has been observed in the germinal vesicle of animals that where new cells are developed from one spot, "the granules composing its periphery are first evolved so as to form a ring of minute vesicles; this ring is afterwards pushed out by a later-formed ring developed from a circle of granules nearer the centre, and this in its turn by another ring. The cells first generated subsequently dissolve away again, their space being occupied by those formed at a later period, which in their turn give place to others; and those last evolved, which originate in the *centre* of the nucleus are the only permanent ones." He then proceeds to show that in the lowest *cryptogamia*, reproduction differs but little from the ordinary mode of multiplication by cells; while in the higher form of this division, "the first nisus is towards the development of a foliaceous expansion, the whole of which remains as the *permanent frond* constituting the perfect plant." In the highest *cryptogamia*, as the ferns, the structure developed from the peripheral cells is merely *temporary*, and the *permanent* portions of the plant originate from some of the interior granules. In the phanerogamic plants the *permanent structures* are only evolved from the central granules, "the peripheral portion soon disappears." In animals "the earliest *nisus* of the development of the germs is the extension of the periphery of the mass of cells first produced, into a membrane which incloses the yelk. An additional layer is subsequently formed internally to this, proceeding from a part of the germinal mass nearer to its centre. The sac thus formed is the simplest condition of a *stomach*, the most characteristic structural distinction between plants and animals. In the radiata the whole of the first-formed structure is *permanent*; the exterior layer constituting the permanent envelope of the body, and the interior the lining of the stomach." In the articulata and mollusca, the parts developed from the peripheral cells still continue a permanent portion, but the organs of *animal* life originate from the central cells of the germinal mass. In the vertebrata the whole of the first-formed structure is *completely transitory* in its character, like the cotyledons of plants. "All the organs of *animal life* which in this group attain so high a development, are developed from the *central* part only of the *nucleus* of the *central* cell of the germinal mass. This is the latest portion of the whole to undergo evolution; but to its development, the evolution of all the rest is subservient." The generalization deduced by the author from the various facts collected on this subject we shall give in his own words:

"Thus when we ascend the scale of being, in either of the two organized kingdoms, we observe the principle of specialization remarkably illustrated, in the development of the germ into the perfect structure. In the lowest of each kind the first-formed membranous expansion has the same character throughout, and the whole enters into the fully-developed structure. In higher grades, the whole remains, but the organs evolved from the centre have evidently the most elevated character. In the highest none but the most central portion is persistent; the remainder forms organs of a temporary and subservient nature." (p. xi.)

We perceive that Dr. Carpenter believes that *acquired peculiarities* under certain limitations may reappear or descend to offspring; and at the end of the fourteenth chapter we find him stating that "no one who has sufficient opportunity of observation, can doubt that the intellectual faculties, which have been developed by cultivation, are generally transmitted to the offspring in an improved state; so that the descendants of a line of educated ancestors will probably have a much higher capacity for instruction than the child that springs from an illiterate race." This doctrine, which many recent observations, particularly those of Mr. Knight, strongly corroborate, is one to which a benevolent and liberal mind naturally leans: we both hope and believe in its soundness.

In leaving this work we again express our unqualified praise of the manner in which the various subjects which it contains have been treated by the author. We know of no work on the subjects of which it treats, which approaches it either in extent and variety of information, or in clearness and precision of thought. We conclude with the following extract from the preface, which we believe truly to represent at once the character of the work and of the author's mind:

"The author has only to add that, in a work comprehending so large a mass of details, many of which he has not had the opportunity of verifying for himself, it can scarcely be expected that no errors should exist. He has endeavoured in every instance to select, amongst conflicting statements, that which seemed most conformable to known principles, and was supported by the authority which was to his own mind most satisfactory. He will always, however, be open to correction, and will gladly avail himself of any that may be offered him. *Truth* is his only object; and, even if his own doctrines should be overthrown by more extended researches, he will rejoice in their demolition as he would in that of any other error. The character of the true philosopher as described by Schiller, one who has *always loved truth better than his system,* will ever, he trusts, be the goal of his intellectual ambition." (p. xii.)

